# Screening and Selection of Antibiotics for Enhanced Production of Astaxanthin by *Haematococcus lacustris*

**DOI:** 10.3390/life14080977

**Published:** 2024-08-02

**Authors:** Vijay Rayamajhi, Huijeong Byeon, Yunji An, Taesoo Kim, Jihyun Lee, JongDae Lee, KwangSoo Lee, ChulHyun Kim, HyunWoung Shin, SangMok Jung

**Affiliations:** 1 Department of Biology, Soonchunhyang University, Asan 31538, Chungcheongnam-do, Republic of Korea; rayamajhivijay8@gmail.com (V.R.); reasyeas@gmail.com (T.K.); 2 Korea Fisheries Resources Agency East Sea Branch, Samho-ro, Buk-gu, Pohang 37601, Gyungsangbuk-do, Republic of Korea; 3 Department of Environmental Health Science, Soonchunhyang University, Asan 31538, Chungcheongnam-do, Republic of Korea; 4 Department of Sports Science, Soonchunhyang University, Asan 31538, Chungcheongnam-do, Republic of Korea; 5 Department of Sports Medicine, Soonchunhyang University, Asan 31538, Chungcheongnam-do, Republic of Korea; 6 AlgaeBio, Inc., Asan 31459, Chungcheongnam-do, Republic of Korea; 7 Research Institute for Basic Science, Soonchunhyang University, Asan 31538, Chungcheongnam-do, Republic of Korea

**Keywords:** *Haematococcus lacustris*, astaxanthin, ampicillin, penicillin, chloramphenicol

## Abstract

*Haematococcus lacustris* (Girod-Chantrans) Rostafinski (Chlorophyta) is the richest microalgal source of astaxanthin. Natural astaxanthin from *H. lacustris* has been widely studied and used for commercial production worldwide. In this study, we examined the effects of 11 antibiotics (dihydrostreptomycin sulphate, neomycin, chloramphenicol, penicillin, streptomycin, ampicillin, kanamycin, gentamycin, hygromycin B, tetracycline, and paromomycin) on the biomass dry weight, growth, and astaxanthin yield of *H. lacustris* using Jaworski’s medium without a nitrogen source. Astaxanthin content in *H. lacustris* was improved in the presence of ampicillin (0.25 g/L, 0.5 g/L, 1 g/L), chloramphenicol (0.25 g/L), and penicillin (0.25 g/L, 0.5 g/L, 1 g/L) in comparison to the control on day 15. The greatest increase in astaxanthin content on day 15 (6.69-fold) was obtained with the addition of penicillin (0.5 g/L) in comparison to the control. Similarly, on day 15, the cell numbers were also the highest for the *H. lacustris* culture grown with the addition of penicillin (0.5 g/L).

## 1. Introduction

Microalgae are unicellular photosynthetic microorganisms that are widely found in both freshwater and saltwater. They belong to several kingdoms, including Bacteria, Protozoa, Chromista, and Plantae [1]. One of the most potent antioxidants known, astaxanthin, has garnered a lot of interest in the biotechnological, scientific, and commercial fields due to its important beneficial biofunctional properties for human nutrition and health. By 2027, the market value of astaxanthin is expected to reach USD 3.4 billion [2]. Astaxanthin, a red xanthophyll carotenoid, is found naturally in fungi, crustaceans, algae, fish, and bacteria [3,4]. The demand for natural astaxanthin for cosmetic and nutraceutical products derived from sustainable and natural sources, like the microalga *Haematococcus lacustris* (Girod-Chantrans) Rostafinski (Chlorophyta), is growing, despite the fact that astaxanthin is produced synthetically for commercial uses [5]. Even though they possess the same molecular formula, 75% of the molecules in natural and synthetic astaxanthin have different configurations [6]. In comparison to natural astaxanthin, synthetic astaxanthin exhibits 20 times less antioxidant properties. In the aquaculture and poultry industries, synthetic astaxanthin is primarily utilized as a source of pigment and is not allowed for human consumption [7]. Natural astaxanthin has a lower environmental impact and is safer than synthetic astaxanthin. The superior antioxidant properties of natural astaxanthin as compared to synthetic astaxanthin might be due to the differences in the stereochemistry and chemical structures. Natural astaxanthin possesses superior water solubility properties than synthetic astaxanthin since all synthetic forms exist in the *trans* form, but 10% of natural astaxanthin exists in the *cis* form [8]. Natural astaxanthin is accepted by the United States Food and Drug Administration (USFDA) as generally recognized as safe (GRAS) [4]. *Haematococcus lacustris* is a freshwater microalga consisting of spherical green biflagellate cells ~30 μm in diameter, belonging to class Chlorophyceae, order Volvocales, and family Haematococcaceae [9], and is considered the richest microalgal source of astaxanthin, with accumulation of up to 5%–6% dry weight (DW) under environmentally ill-favored conditions derived from nitrogen/phosphorous deficiency, salt stress, and high light intensity [10]. The life cycle of *H. lacustris* involves four distinct cellular morphologies; the green vegetative phase consists of macrozooids (zoospores), microzooids, and palmella, and the astaxanthin-accumulated, red, nonmotile, encysted phase consists of haematocysts (aplanospores). Macrozooids (zoospores) are pear-shaped, spherical, or ellipsoidal cells with a size of 8–20 μm and possessing two flagella of the same length. Macrozooids may divide by mitosis into 2–32 daughter cells. On the onset of unfavorable culture or environmental conditions, macrozooids increase their cell size, lose their flagella, turn into stationary “palmella”, and become vegetative cells in a resting state. With the continuity of stress such as high salinity, nutrient deprivation, and high light irradiance, “palmella” develops into “aplanospores”, which are asexual and resistant to drastic environmental changes. On maturity, these “aplanospores” accumulate large amounts of astaxanthin [11]. Commercial production of natural astaxanthin from *H. lacustris* has been studied and applied worldwide, and there is a potential economic opportunity, especially for nations where the climate is suitable for its cultivation [12]. Nitrogen is one of the necessary elements that influence the enzymatic activity and cell growth of *H. lacustris.* Nitrogen deprivation can stimulate astaxanthin biosynthesis [13,14].

The development of microalgae technologies is compromised due to the contamination of microalgae cultures [1]. Microalgal cultivation is affected by biological contamination, which can lead to culture crashes and reduced yields. The most common biological contaminants are grazers, fungi, photosynthetic microorganisms, bacteria, viruses, and parasites [15]. These contaminants harm culture growth, reduce production, and occasionally target the final product or cause the complete loss of microorganisms [16]. Some antibiotics such as amphotericin B and cephalosporin are able to induce the formation of astaxanthin as an indicator of unfavorable conditions [17]. Dihydrostreptomycin sulphate, neomycin, kanamycin, gentamicin, and streptomycin are aminoglycoside antibiotics, which have bactericidal properties mediated via the disruption of protein synthesis by binding to the 30S ribosomal subunit (or the small 16S rRNA component in the case of streptomycin) [18,19,20,21,22,23,24]. Chloramphenicol is a synthetic broad-spectrum antibiotic with a bacteriostatic mode of action [25]. Penicillin is a β-lactam antibiotic with bactericidal properties [26]. Ampicillin is a broad-spectrum antibiotic with antibacterial activity against anaerobic, Gram-positive, and Gram-negative bacteria [27]. Hygromycin B is an aminoglycoside antibiotic that is effective against both eukaryotic and prokaryotic cells and has been shown to control fungal cell growth [28]. Tetracycline is a broad-spectrum antibiotic agent effective against Gram-negative and Gram-positive bacteria, *Chlamydia*, *Mycoplasma*, protozoan parasites, and *Rickettsia* [29]. Paromomycin is an antimicrobial agent used in the treatment of parasitic infections [30]. Although antibiotics may represent a less costly alternative to conventional sterilization methods, their use is not recommended for products that are consumed as food or food supplements. However, astaxanthin produced through the use of antibiotics can be used in cosmetics and some pharmaceutical applications [15].

We performed this study as we were facing severe contamination problems in *H. lacustris* cultures due to unknown contaminants in our previously developed hybrid open–closed cultivation system [7]. In this study, we examined the effects of 11 antibiotics (dihydrostreptomycin sulphate, neomycin, chloramphenicol, penicillin, streptomycin, ampicillin, kanamycin, gentamycin, hygromycin B, tetracycline, and paromomycin) on the biomass dry weight, growth, and astaxanthin yields of *H. lacustris* using Jaworski’s medium (JM) without a nitrogen source in the laboratory, with the aim of inhibiting the growth of contaminants and improving astaxanthin yields from *H. lacustris*. 

## 2. Materials and Methods

### 2.1. Microalgae and Culture Conditions

We obtained *H. lacustris* (LIMS-PS-1354) from the Library of Marine Samples at the Korea Institute of Ocean Science and Technology (Geoje, Republic of Korea). *Haematococcus lacustris* was cultured and maintained under a photon flux density of 37 µmol m^−2^ s^−1^ at 21 °C ± 1 °C under a 12 h/12 h light/dark cycle in JM. For the experiment, *H. lacustris* was cultured at 16 °C ± 2 °C under a photon flux density of 40 µmol m^−2^ s^−1^ in JM without a nitrogen source, which was prepared as described in our recent studies [2,7,31]. Fluorescent light intensity was measured with a light meter (Fluke-941; Fluke Corp., Everett, WA, USA). The experiments were carried out in 40 mL culture flasks with 30 mL of growth medium and 10 mL of seed culture. A 4-day-old culture was used as the initial inoculum for seed culture. All experiments were replicated three times. In the experiments, the 11 antibiotics were added to *H. lacustris* cultures at varying concentrations of 0 g/L, 0.25 g/L (i.e., 250 ppm), 0.5 g/L (500 ppm), and 1 g/L (1000 ppm). Hygromycin B and neomycin were supplied from Sigma-Aldrich (St. Louis, MO, USA) as standard stock solutions of 50 and 10 mg/mL, respectively. Other antibiotic stock solutions of 100 mg/mL were prepared by weighing and dissolving the antibiotics in autoclaved water using 0.2 μm nylon syringe filters (Hyundai Micro, Seoul, Republic of Korea) with a diameter of 13 mm.

### 2.2. Chemicals and Reagents

Hygromycin B from *Streptomyces hygroscopicus*, gentamycin sulphate, penicillin G sodium salt, dihydrostreptomycin sesquisulphate, kanamycin sulphate from *Streptomyces kanamyceticus*, neomycin solution (10 mg/mL), paromomycin, tetracycline, biotin, and thiamine hydrochloride were obtained from Sigma-Aldrich (St. Louis, MO, USA). Streptomycin, chloramphenicol, potassium nitrate, dibasic anhydrous potassium phosphate, dibasic anhydrous sodium phosphate, sodium bicarbonate, and cyanocobalamin were obtained from Daejung Chemicals & Metals (Siheung, Gyronhhi-do, Republic of Korea). Ampicillin sodium salt was obtained from Fisher Bioreagents (Fair Lawn, NJ, USA). Ammonium molybdate tetrahydrate was obtained from Duksan Reagents (Asan, Gyeonggi-do, Republic of Korea). All chemicals were of biograde, unless otherwise indicated.

### 2.3. Biomass Dry Weight, Cell Number, and Astaxanthin Accumulation Analyses

To evaluate the impact of antibiotics on the biomass dry weight of *H. lacustris* cultures, we cultivated 8 distinct samples of *H. lacustris* at varying antibiotic concentrations. These samples were the control, ampicillin (0.25 g/L, 0.5 g/L, 1 g/L), penicillin (0.25 g/L, 0.5 g/L, 1 g/L), and chloramphenicol (0.25 g/L). These samples were selected because the astaxanthin contents of the *H. lacustris* cultures grown with the addition of these antibiotics were higher than the control on day 15. All these samples were grown in a volume of 20 mL (15 mL JM without nitrogen source and 5 mL of 4-day old seed culture) with an initial cell number of 1.6 ± 0.16 × 10^4^ cells/mL each at 16 °C ± 2 °C under a photon flux density of 40 µmol m^−2^ s^−1^ for 15 days. Later, the 15-day samples were centrifuged at 3800× *g* for 10 min using Hanil Science Medical Centrifuge (FLETA5) from Hanil Scientific Inc. (Gimpo, Republic of Korea). The supernatants were discarded, and the pellets were dried at 100 °C using a pre-weighed aluminum dish for 24 h until a constant weight was maintained. The pellets were then cooled to room temperature and weighed. 

Cell numbers were estimated using a light microscope with a 20× objective lens (BX53; Olympus, Tokyo, Japan) and a hemocytometer with an improved Neubauer chamber (Marienfeld Superior, Lauda-Königshofen, Germany). Astaxanthin accumulation by all the *H. lacustris* cultures used in this experiment was estimated according to protocols used in our recent studies [2,7]. For the estimation of astaxanthin content, 10 mL of samples from the 15-day culture were taken in conical tubes and centrifuged for 10 min at 1700× *g*. The pellets obtained were then treated with 5% (*w*/*v*) KOH and further diluted with 30% (*v*/*v*) methanol. The chlorophyll was degraded at 70 °C for 10 min, and the samples were centrifuged at 3500× *g* for 10 min. A total of 100 µL of glacial acetic acid was used to acidify the supernatants. Following this, 5 mL of dimethyl sulfoxide (DMSO) was added to the solution and boiled at 70 °C for 15 min. Furthermore, a final centrifugation was performed, the supernatants were pooled, and the astaxanthin content was determined using a spectrophotometer by measuring the absorbance at 490 nm as follows [32]:

Astaxanthin (mg/L) = [4.5 × A_490_ × (V*_a_* ÷ V*_b_*)]

where A_490_ is the absorbance of the supernatant at 490 nm; V*_a_* and V*_b_* are the volumes of DMSO and microalga samples, respectively.

### 2.4. Statistical Analyses

Cell numbers, DW, and astaxanthin yield data are presented as mean ± standard deviation. Means were compared using one-way analysis of variance (ANOVA), followed by Tukey’s multiple comparison test using SPSS v22 (IBM Corp., Armonk, NY, USA). In all the analyses, the significance was evaluated at a level of *p* < 0.001.

## 3. Results

### 3.1. Effects of Antibiotics on the Cell Numbers and Astaxanthin Content of H. lacustris on Day 15

**Figure 1:**
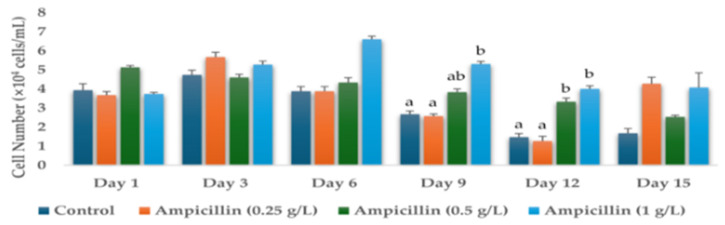
Cell numbers of *H. lacustris* grown in JM without nitrogen source (Day 1, Day 3, Day 6, Day 9, Day 12, Day 15 with the use of ampicillin (0.25 g/L, 0.5 g/L, 1 g/L). Data are presented as mean ± standard deviation. Lowercase letters indicate significant differences (*p* < 0.001).

**Figure 2:**
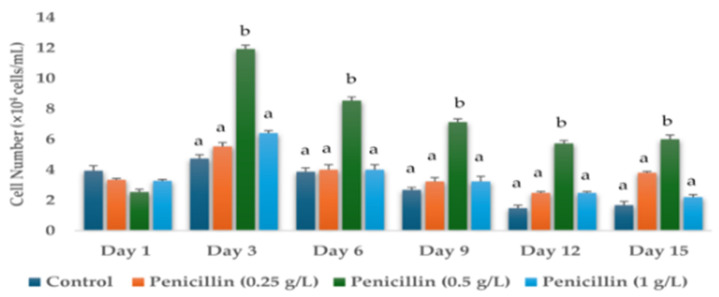
Cell numbers of *H. lacustris* grown in JM without nitrogen source (Day 1, Day 3, Day 6, Day 9, Day 12, Day 15 with the use of penicillin (0.25 g/L, 0.5 g/L, 1 g/L). Data are presented as mean ± standard deviation. Lowercase letters indicate significant differences (*p* < 0.001).

Cell numbers were determined on days 1, 3, 6, 9, 12, and 15 of the *H. lacustris* cultures grown in the presence of different concentrations of antibiotics—0 g/L (control), 0.25 g/L, 0.5 g/L, and 1 g/L. Cell numbers (days 1, 3, 6, 9, 12, and 15) of the *H. lacustris* cultures grown in the presence of hygromycin B, ampicillin, neomycin, paromomycin, tetracycline, kanamycin, chloramphenicol, streptomycin, dihydrostreptomycin sulphate, penicillin, and gentamicin are shown in Tables S1–S11. Significant results were obtained for the cell numbers of *H. lacustris* cultures grown with the addition of ampicillin and penicillin (Figure 1 and Figure 2). The initial cell number used for this experiment on Day 0 was 3.93 ± 0.34 × 10^4^ cells/mL, which was the same as the control for all the samples.

The inhibition of cell growth was observed for the culture samples of *H. lacustris* with the addition of neomycin (0.25 g/L, 0.5 g/L, 1 g/L), paromomycin (0.25 g/L, 0.5 g/L, 1 g/L), kanamycin (0.5 g/L, 1 g/L), chloramphenicol (0.25 g/L, 0.5 g/L, 1 g/L), streptomycin (0.25 g/L, 0.5 g/L, 1 g/L), dihydrostreptomycin sulfate (0.25 g/L, 0.5 g/L, 1 g/L), and gentamycin (0.25 g/L, 0.5 g/L, 1 g/L), even on day 1, as the cell number reading showed a drastic decrease in their counts, although the same amount of cell number was used for the inoculation of all the experimental culture samples, including the control, on the initial day (Day 0). All cells died by day 15 in the cultures grown in the presence of chloramphenicol (1 g/L), dihydrostreptomycin sulphate (0.25 g/L, 0.5 g/L, 1 g/L), gentamycin (1 g/L), hygromycin B (0.25 g/L, 0.5 g/L, 1 g/L), kanamycin (0.25 g/L, 0.5 g/L, 1 g/L), neomycin (0.25 g/L, 0.5 g/L, 1 g/L), paromomycin (0.5 g/L), and streptomycin (0.25 g/L, 0.5 g/L, 1 g/L). 

**Figure 3:**
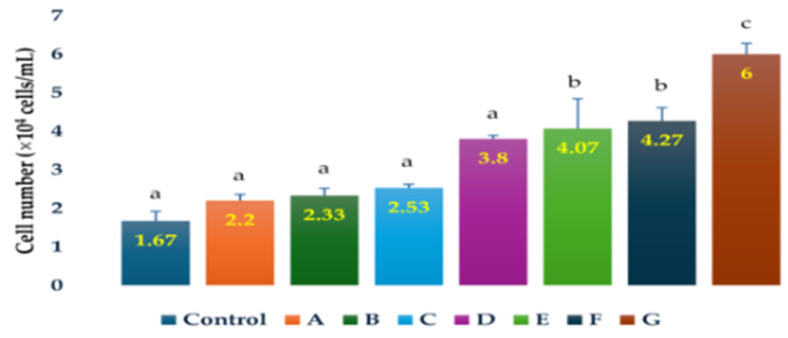
Comparison of cell numbers of *H. lacustris* culture samples on day 15 grown in JM without a nitrogen source with control, A-addition of penicillin (1 g/L), B-addition of chloramphenicol (0.25 g/L), C-addition of ampicillin (0.5 g/L), D-addition of penicillin (0.25 g/L), E-addition of ampicillin (1 g/L), F-addition of ampicillin (0.25 g/L), and G-addition of penicillin (0.5 g/L). Mean values that do not share the same letter are significantly different at *p* < 0.001.

The cell numbers of *H. lacustris* on day 15 were higher in the cultures grown in the presence of ampicillin (0.25 g/L, 0.5 g/L, 1 g/L; 2.55-, 1.51-, and 2.43-fold, respectively), chloramphenicol (0.25 g/L; 1.39-fold), penicillin (0.25 g/L, 0.5 g/L, 1 g/L; 2.27-, 3.59- and 1.31-fold, respectively), and tetracycline (1 g/L; 1.23-fold) than those of the control (Figure 3). The greatest increase in cell number compared to the control (3.59-fold) was observed in the cultures grown in the presence of 0.5 g/L penicillin.

**Figure 4:**
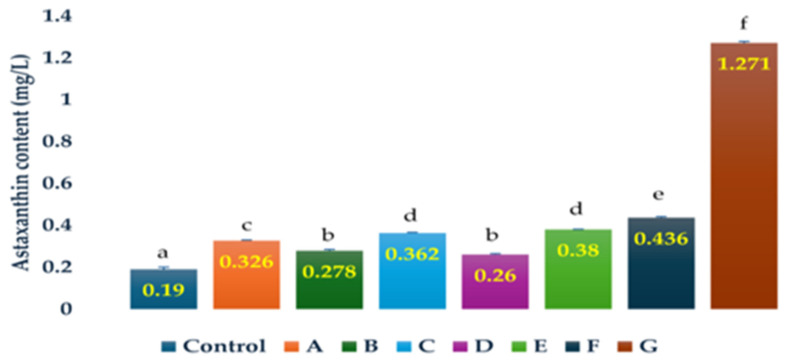
Comparison of astaxanthin accumulation by *H. lacustris* culture samples on day 15 grown in JM without a nitrogen source with control, A-addition of penicillin (1 g/L), B-addition of chloramphenicol (0.25 g/L), C-addition of ampicillin (0.5 g/L), D-addition of penicillin (0.25 g/L), E-addition of ampicillin (1 g/L), F-addition of ampicillin (0.25 g/L), and G-addition of penicillin (0.5 g/L). Mean values that do not share the same letter are significantly different at *p* < 0.001.

The astaxanthin contents of *H. lacustris* on day 15 were higher than in the control in cultures grown in the presence of ampicillin (0.25 g/L, 0.5 g/L, 1 g/L; 2.29-, 1.90- and 2.0-fold, respectively), chloramphenicol (0.25 g/L; 1.46-fold), and penicillin (0.25 g/L, 0.5 g/L, 1 g/L; 1.38-, 6.69-, and 1.71-fold, respectively) (Figure 4). The greatest increase in astaxanthin content compared to the control (6.69-fold) was observed in cultures grown in the presence of 0.5 g/L penicillin. The readings for the astaxanthin contents of all the experimental samples on day 15 are shown in Tables S1–S11.

We have summarized our results on the effects of antibiotics on the cell number and astaxanthin accumulation of *H. lacustris* on Day 15 in Table 1.

In comparison, it was observed that the astaxanthin contents of *H. lacustris* increased with the increase in cell numbers. *H. lacustris* cultures with higher cell numbers also showed higher astaxanthin contents. Among the results of eight *H. lacustris* culture samples compared, there was a general tendency for a gradual increase in astaxanthin contents with the increase in cell number, except for the *H. lacustris* culture grown with the addition of penicillin (1 g/L) and penicillin (0.25 g/L). This could be due to the irregular size of *H. lacustris* cells. Although astaxanthin contents are dependent on the cell numbers, the size of the cells also could determine the amount of astaxanthin, as some cells might store higher contents of astaxanthin, whereas others might store lower contents of astaxanthin. 

### 3.2. Effects of Antibiotics on the Biomass Dry Weight of H. lacustris on Day-15

**Figure 5:**
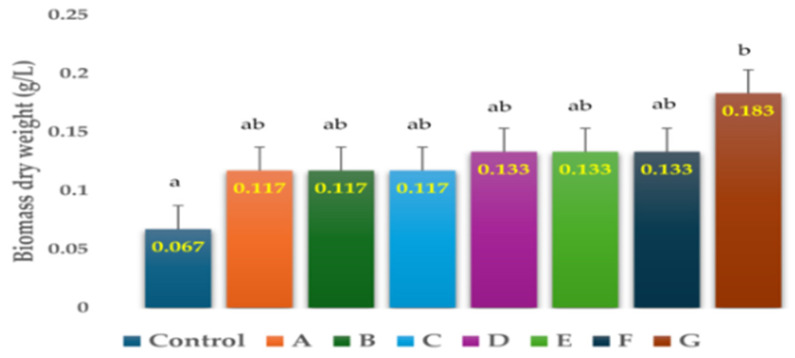
Comparison of biomass dry weight of *H. lacustris* culture samples on day 15 grown in JM without a nitrogen source with control, A-addition of penicillin (1 g/L), B-addition of chloramphenicol (0.25 g/L), C-addition of ampicillin (0.5 g/L), D-addition of penicillin (0.25 g/L), E-addition of ampicillin (1 g/L), F-addition of ampicillin (0.25 g/L), and G-addition of penicillin (0.5 g/L). Mean values that do not share the same letter are significantly different at *p* < 0.001.

To compare the biomass dry weight, we selected the *H. lacustris* culture samples that showed higher astaxanthin contents than the control from our previous experiment. Therefore, we cultivated 20 mL each of eight samples of *H. lacustris* culture with the initial cell number of 1.6 ± 0.16 × 10^4^ cells/mL under the same experimental conditions. The results of the effects of antibiotics on the biomass dry weight of eight samples of *H. lacustris* culture are shown in Table 2 and Figure 5.

The biomass dry weights of *H. lacustris* on day 15 were higher in the cultures grown in the presence of ampicillin (0.25 g/L, 0.5 g/L, 1 g/L; 1.98-, 1.74- and 1.98-fold, respectively), chloramphenicol (0.25 g/L; 1.74-fold) and penicillin (0.25 g/L, 0.5 g/L, 1 g/L; 1.98-, 2.73-, and 1.74-fold, respectively) than in the control (Figure 5). The greatest increase in biomass dry weight compared to the control (2.73-fold) was observed in cultures grown in the presence of 0.5 g/L penicillin. The results of the increase in biomass dry weight of these selected *H. lacustris* culture samples were similar to the results of the increase in cell numbers from our previous experiment for the same specific *H. lacustris* cultures. *H. lacustris* cultures with higher cell numbers showed higher biomass dry weight, and *H. lacustris* cultures with lower cell numbers showed lower biomass dry weight. Overall, the biomass dry weight results were comparatively lower as the initial inoculum of cell numbers of *H. lacustris* used for this study was lower, which could be increased by selecting a higher initial inoculation cell number of *H. lacustris*. 

## 4. Discussion

The production of carotenoids can be stimulated in microalgae through the antibiotic-induced moderate stress levels [33], whereas higher levels of antibiotics decrease the contents of total carotenoids [34]. There is little evidence for the enhanced carotenoid accumulation in microalgae using antibiotics. In *Raphidocelis subcaitata,* there was significant increase in the carotenoid contents with the use of erythromycin at the concentrations of 0.001 mg/L and 0.003 mg/L [34]. Similarly, the carotenoid production was increased up to 32% in *Auxenochlorella protothecoides*, *Tetradesmus obliquus*, and *Chlamydomonas acidophila* using a combination of seven antibiotics (trimethoprim, clarithromycin, metronidazole, ofloxacin, ciprofloxacin, sulfamethoxazole, and erythromycin) at a concentration of 100 μg/L [33]. The results of this study showed that *H. lacustris* cells could not survive in the presence of streptomycin and gentamycin. These two aminoglycoside antibiotics inhibit plastid protein synthesis by entering the algal chloroplast and targeting the 30S ribosomal subunit, thereby hindering protein synthesis and cell growth [35]. Antibiotic treatments have been used to effectively remove bacterial contamination from microalgal cultures [36]. Previous studies have obtained optimum concentrations of antibiotics for the axenic culture of some marine microalgae, e.g., 500 ppm of neomycin for *Isochrysis galbana* and *Heterosigma akashiwo*, 500 ppm of chloramphenicol for *Cyclotella didymus*, 2000 ppm of dihydrostreptomycin for *Chlorella ellipsoidea*, and 6000 ppm of neomycin and dihydrostreptomycin sulphate for *Thalassiosira allenii* [37]. The productivity of an unsterilized open system culture was enhanced by using specific antibiotics, which reduced contamination. Biological contaminants were shown to impede the cost-effective production of bulk microalgal biomass volumes [38]. 

Antibiotics are detrimental to both microalgae and bacteria. Specific microalgae die in the presence of low concentrations of certain types of antibiotics. Bacteria were successfully removed from cultures of *Chrysotila roscoffensis* using a cocktail composed of geneticin, gentamycin, streptomycin, ampicillin, and kanamycin [39]. The use of commercial fertilizers can also increase astaxanthin content and cell numbers in *H. lacustris* [40]. The use of antibiotics has recently increased globally for the treatment and prevention of bacterial contamination. As animals and humans cannot completely metabolize antibiotics, their residues can lead to problems, such as the appearance of antibiotic-resistant bacteria and antibiotic resistance genes [41]. However, *H. lacustris* is also efficient in the removal of antibiotics [41,42,43]. The efficacy and toxicity of the antibiotic treatment are determined by the concentration and exposure period and vary between microalgal and bacterial species. There is a rapid reduction in viable bacteria after 48 h of antibiotic treatment in microalgal cultures. Penicillin is bactericidal as it interferes with the bacterial cell wall formation, whereas chloramphenicol and tetracycline are bacteriostatic, as they do not necessarily kill bacteria but interfere with their metabolism [44]. Algal cultures are often contaminated by fungi in addition to bacteria. Antibiotics have been used for the removal of bacterial contamination from the cultures of microalgae [36,39]. Antibiotic treatments are performed to reduce bacterial contamination, which will let the inoculum of microalgal cells grow without viable bacteria. The use of antibiotics also prevents fungal contamination in microalgal culture. However, apart from the use of antibiotics, other methods are also applied for controlling the growth of undesired microorganisms in microalgal cultures. These methods include incubating the microalgal culture at a temperature outside the growth range of contaminant microorganisms, growing the microalgal culture at a pH where the growth of biological contaminants is suppressed but the growth of viable microalgae is maintained, using pulsed electric fields to damage selective cells in the culture, and using other chemical agents such as pesticides, aldehydes, fungicides, salts, ammonia, peroxides, etc. [44]. A cocktail of antibiotics, including carbendazim, ampicillin, and cefotaxime, was used to remove both bacterial and fungal contamination in cultures of *Chlamydomonas reinhardtii* [45]. Eukaryotic microalgae may be less or not susceptible to aminopenicillin antibiotics [46]. With the addition of antibiotics, there are various chances of enhancing microalgal growth. The added antibiotics can be biodegraded or decomposed by microalgae and utilized as electron donors and carbon sources, thus promoting the growth of microalgae [47]. In a study by [48], antibiotics ciprofloxacin and amoxicillin were utilized as carbon sources for the growth of *Chlorella vulgaris*. In addition, ref. [49] had engineered *E. coli* strains capable of utilizing penicillin as the sole carbon source. Sulfadiazine (30 mg/L) and sulfamerazine (90 mg/L) served as carbon sources for the promotion of growth in *Chlorella vulgaris* [50].

Among the eleven antibiotics used in this study, our results indicated that the supplementation of only three of these antibiotics—ampicillin, chloramphenicol, and penicillin—enhanced the astaxanthin content in *H. lacustris* culture on day 15. Similarly, the cell numbers were higher than the control on day 15 for only those *H. lacustris* cultures grown with the supplementation of four antibiotics, ampicillin, chloramphenicol, penicillin, and tetracycline. The use of penicillin (0.5 g/L) was clearly the best choice of antibiotics for the culture of *H. lacustris* as the results indicated that its addition significantly enhanced the cell numbers, biomass dry weight, and astaxanthin contents of *H. lacustris* culture on day 15. *H. lacustris* was sensitive to hygromycin B, neomycin, paromomycin, kanamycin, streptomycin, dihydrostreptomycin sulfate, and gentamycin. A more detailed study on the effects of the combination of multiple antibiotics in the future would be helpful for optimizing antibiotic usage during the mass cultivation of *H. lacustris* for commercial production of astaxanthin. Interestingly, the cells of *H. lacustris* culture survived with the use of tetracycline, but the biosynthesis of astaxanthin was inhibited. Tetracyclines belong to the group of tetracyclic antibiotics and possess anti-inflammatory effects [51]. According to [52], carotenoid accumulation of *H. lacustris* was inhibited in the palmella stage with the use of non-steroidal anti-inflammatory drugs. Therefore, further investigation is necessary to decipher the mechanism behind the inhibition of astaxanthin accumulation by tetracycline in *H. lacustris*. Although the astaxanthin yields were improved in *H. lacustris* by the addition of specific antibiotics in this study, the addition of antibiotics to *H. lacustris* for the enhancement of astaxanthin is not a sustainably preferable method for commercial benefits because the antibiotics are expensive. Therefore, the use of antibiotics should instead be limited to obtaining axenic *H. lacustris* culture, which would be economical. The axenic culture thus obtained should be maintained by periodic subculture, freeze drying, and cryopreservation [44]. Further research is still required to determine the toxicity of these antibiotics to living organisms and the environment. Moreover, we will conduct further research on the characterization and control of specific contaminants isolated from *H. lacustris* culture in the future. There is a need for safer alternatives for the removal of biological contamination from microalgal cultures. The bioremediation of antibiotics could also be introduced in parallel to neutralize the harmful effects of residual antibiotics.

## Figures and Tables

**Table 1:** Comparison of cell numbers and astaxanthin of *H. lacustris* culture grown in JM without nitrogen source with the addition of penicillin (1 g/L), chloramphenicol (0.25 g/L), ampicillin (0.5 g/L), penicillin (0.25 g/L), ampicillin (1 g/L), ampicillin (0.25 g/L), penicillin (0.5 g/L), and control on day 15. Data are presented as mean ± standard deviation. Lowercase letters indicate significant differences (*p* < 0.001).

*H. lacustris* Culture	Day 15 Cell Number (×10^4^ Cells/mL)	Day 15 Astaxanthin Content (mg/L)
Control	1.67 ± 0.25 ^a^	0.190 ± 0.010 ^a^
Penicillin (1 g/L)	2.20 ± 0.16 ^a^	0.326 ± 0.004 ^c^
Chloramphenicol (0.25 g/L)	2.33 ± 0.19 ^a^	0.278 ± 0.006 ^b^
Ampicillin (0.5 g/L)	2.53 ± 0.09 ^a^	0.362 ± 0.003 ^d^
Penicllin (0.25 g/L)	3.8 ± 0.09 ^a^	0.26 ± 0.003 ^b^
Ampicillin (1 g/L)	4.07 ± 0.77 ^b^	0.380 ± 0.001 ^d^
Ampicillin (0.25 g/L)	4.27 ± 0.34 ^b^	0.436 ± 0.005 ^e^
Penicillin (0.5 g/L)	6.00 ± 0.28 ^c^	1.271 ± 0.007 ^f^

**Table 2:** Comparison of biomass dry weight of *H. lacustris* culture grown in JM without nitrogen source with the addition of penicillin (1 g/L), chloramphenicol (0.25 g/L), ampicillin (0.5 g/L), penicillin (0.25 g/L), ampicillin (1 g/L), ampicillin (0.25 g/L), penicillin (0.5 g/L), and control on day 15. Data are presented as mean ± standard deviation. Lowercase letters indicate significant differences (*p* < 0.001).

*H. Lacustris* Culture	Day 15 Biomass Dry Weight (g/L)
Control	0.067 ± 0.02 ^a^
Penicillin (1 g/L)	0.117 ± 0.02 ^ab^
Chloramphenicol (0.25 g/L)	0.117 ± 0.02 ^ab^
Ampicillin (0.5 g/L)	0.117 ± 0.02 ^ab^
Penicillin (0.25 g/L)	0.133 ± 0.02 ^ab^
Ampicillin (1 g/L)	0.133 ± 0.02 ^ab^
Ampicillin (0.25 g/L)	0.133 ± 0.02 ^ab^
Penicillin (0.5 g/L)	0.183 ± 0.02 ^b^

## Data Availability

Data are contained within the article.

## Supplementary Materials

The following supporting information can be downloaded at: https://www.mdpi.com/article/10.3390/life14080977/s1, Table S1. Cell numbers of *H. lacustris* grown in JM without nitrogen source (Day 1, Day 3, Day 6, Day 9, Day 12, Day 15) and astaxanthin content (Day 15) with the use of Hygromycin B (0.25 g/L, 0.5 g/L, 1 g/L); Table S2. Cell numbers of *H. lacustris* grown in JM without nitrogen source (Day 1, Day 3, Day 6, Day 9, Day 12, Day 15) and astaxanthin content (Day 15) with the use of Ampicillin (0.25 g/L, 0.5 g/L, 1 g/L); Table S3. Cell numbers of *H. lacustris* grown in JM without nitrogen source (Day 1, Day 3, Day 6, Day 9, Day 12, Day 15) and astaxanthin content (Day 15) with the use of Neomycin (0.25 g/L, 0.5 g/L, 1 g/L); Table S4. Cell numbers of *H. lacustris* grown in JM without nitrogen source (Day 1, Day 3, Day 6, Day 9, Day 12, Day 15) and astaxanthin content (Day 15), and DW (Day 15) with the use of Paromomycin (0.25 g/L, 0.5 g/L, 1 g/L); Table S5. Cell numbers of *H. lacustris* grown in JM without nitrogen source (Day 1, Day 3, Day 6, Day 9, Day 12, Day 15) and astaxanthin content (Day 15) with the use of Tetracycline (0.25 g/L, 0.5 g/L, 1 g/L); Table S6. Cell numbers of *H. lacustris* grown in JM without nitrogen source (Day 1, Day 3, Day 6, Day 9, Day 12, Day 15) and astaxanthin content (Day 15) with the use of Kanamycin (0.25 g/L, 0.5 g/L, 1 g/L).; Table S7. Cell numbers of *H. lacustris* grown in JM without nitrogen source (Day 1, Day 3, Day 6, Day 9, Day 12, Day 15) and astaxanthin content (Day 15) with the use of Chloramphenicol (0.25 g/L, 0.5 g/L, 1 g/L); Table S8. Cell numbers of *H. lacustris* grown in JM without nitrogen source (Day 1, Day 3, Day 6, Day 9, Day 12, Day 15) and astaxanthin content (Day 15) with the use of Streptomycin (0.25 g/L, 0.5 g/L, 1 g/L); Table S9. Cell numbers of *H. lacustris* grown in JM without nitrogen source (Day 1, Day 3, Day 6, Day 9, Day 12, Day 15) and astaxanthin content (Day 15) with the use of Dihydrostreptomycin sulfate (0.25 g/L, 0.5 g/L, 1 g/L); Table S10. Cell numbers of *H. lacustris* grown in JM without nitrogen source (Day 1, Day 3, Day 6, Day 9, Day 12, Day 15) and astaxanthin content (Day 15) with the use of Penicillin (0.25 g/L, 0.5 g/L, 1 g/L); Table S11. Cell numbers of *H. lacustris* grown in JM without nitrogen source (Day 1, Day 3, Day 6, Day 9, Day 12, Day 15) and astaxanthin content (Day 15) with the use of Gentamycin (0.25 g/L, 0.5 g/L, 1 g/L).
