# Supplementary material for: Screening and Selection of Antibiotics for Enhanced Production of Astaxanthin by Haematococcus lacustris
Source: Life (Basel). 2024 Aug 2;14(8):977. doi: 10.3390/life14080977 (PMC11355620; doi:10.3390/life14080977)
Supplement: Supplementary file 1 [file life-14-00977-s001.zip › life-3131265-supplementary.pdf]

Supplementary Material:

Table S1. Cell numbers of *H. lacustris* grown in JM without nitrogen source (Day 1, Day 3, Day 6, Day 9, Day 12, Day 15) and astaxanthin content (Day 15) with the use of Hygromycin B (0.25 g/L, 0.5 g/L, 1 g/L). Data are presented as mean ± standard deviation. Lowercase letters indicate significant differences ($p < 0.001$).

H. lacustris culture grown with Hygromycin B	Day 1 Cell Number (x 10^4 cells/mL)	Day 3 Cell Number (x 10^4 cells/mL)	Day 6 Cell Number (x 10^4 cells/mL)	Day 9 Cell Number (x 10^4 cells/mL)	Day 12 Cell Number (x 10^4 cells/mL)	Day 15 Cell Number (x 10^4 cells/mL	Day 15 Astaxanthin (mg/L)
Control	3.93 ± 0.34[b]	4.73 ± 0.25[b]	3.87 ± 0.25[b]	2.67 ± 0.17	1.47 ± 0.19	1.67 ± 0.25	0.190 ± 0.010
Hygromycin B (0.25 g/L)	1.67 ± 0.09[ab]	0.33 ± 0.09[a]	0.13 ± 0.19[a]	0.10 ± 0.08	0.07 ± 0.09	0.00 ± 0.00	0.00 ± 0.000
Hygromycin B (0.5 g/L)	1.20 ± 0.16[a]	0.33 ± 0.09[a]	0.00 ± 0.00[a]	0.00 ± 0.00	0.00 ± 0.00	0.00 ± 0.00	0.00 ± 0.000
Hygromycin B (1 g/L)	2.00 ± 0.00[ab]	0.27 ± 0.09[a]	0.00 ± 0.00[a]	0.00 ± 0.00	0.00 ± 0.00	0.00 ± 0.00	0.00 ± 0.000

Table S2. Cell numbers of *H. lacustris* grown in JM without nitrogen source (Day 1, Day 3, Day 6, Day 9, Day 12, Day 15) and astaxanthin content (Day 15) with the use of Ampicillin (0.25 g/L, 0.5 g/L, 1 g/L). Data are presented as mean ± standard deviation. Lowercase letters indicate significant differences ($p < 0.001$).

H. lacustris culture grown with Ampicillin	Day 1 Cell Number (x 10^4 cells/mL)	Day 3 Cell Number (x 10^4 cells/mL)	Day 6 Cell Number (x 10^4 cells/mL)	Day 9 Cell Number (x 10^4 cells/mL)	Day 12 Cell Number (x 10^4 cells/mL)	Day 15 Cell Number (x 10^4 cells/mL	Day 15 Astaxanthin (mg/L)
Control	3.93 ± 0.34	4.73 ± 0.25	3.87 ± 0.25	2.67 ± 0.17[a]	1.47 ± 0.19[a]	1.67 ± 0.25	0.190 ± 0.010[a]
Ampicillin (0.25 g/L)	3.67 ± 0.19	5.67 ± 0.25	3.87 ± 0.25	2.57 ± 0.12[a]	1.27 ± 0.25[a]	4.27 ± 0.34	0.436 ± 0.005[b]
Ampicillin (0.5 g/L)	5.13 ± 0.09	4.60 ± 0.16	4.33 ± 0.25	3.83 ± 0.17[ab]	3.33 ± 0.19[b]	2.53 ± 0.09	0.362 ± 0.003[c]
Ampicillin (1 g/L)	3.73 ± 0.09	5.27 ± 0.19	6.60 ± 0.16	5.30 ± 0.14[b]	4.00 ± 0.16[b]	4.07 ± 0.77	0.380 ± 0.001[c]

Table S3. Cell numbers of *H. lacustris* grown in JM without nitrogen source (Day 1, Day 3, Day 6, Day 9, Day 12, Day 15) and astaxanthin content (Day 15) with the use of Neomycin (0.25 g/L, 0.5 g/L, 1 g/L). Data are presented as mean ± standard deviation.

H. lacustris culture grown with Neomycin	Day 1 Cell Number (x 10^4 cells/mL)	Day 3 Cell Number (x 10^4 cells/mL)	Day 6 Cell Number (x 10^4 cells/mL)	Day 9 Cell Number (x 10^4 cells/mL)	Day 12 Cell Number (x 10^4 cells/mL)	Day 15 Cell Number (x 10^4 cells/mL	Day 15 Astaxanthin (mg/L)

Control	3.93 ± 0.34	4.73 ± 0.25	3.87 ± 0.25	2.67 ± 0.17	1.47 ± 0.19	1.67 ± 0.25	0.190 ± 0.010
Neomycin (0.25 g/L)	0.00 ± 0.000	0.00 ± 0.00	0.00 ± 0.00	0.00 ± 0.00	0.00 ± 0.00	0.00 ± 0.00	0.00 ± 0.000
Neomycin (0.5 g/L)	0.00 ± 0.000	0.07 ± 0.094	0.00 ± 0.00	0.00 ± 0.00	0.00 ± 0.00	0.00 ± 0.00	0.00 ± 0.000
Neomycin (1 g/L)	0.00 ± 0.000	0.13 ± 0.094	0.00 ± 0.00	0.00 ± 0.00	0.00 ± 0.00	0.00 ± 0.00	0.00 ± 0.000

Table S4. Cell numbers of *H. lacustris* grown in JM without nitrogen source (Day 1, Day 3, Day 6, Day 9, Day 12, Day 15) and astaxanthin content (Day 15), and DW (Day 15) with the use of Paromomycin (0.25 g/L, 0.5 g/L, 1 g/L). Data are presented as mean ± standard deviation.

H. lacustris culture grown with Paromomycin	Day 1 Cell Number (x 10^4 cells/mL)	Day 3 Cell Number (x 10^4 cells/mL)	Day 6 Cell Number (x 10^4 cells/mL)	Day 9 Cell Number (x 10^4 cells/mL)	Day 12 Cell Number (x 10^4 cells/mL)	Day 15 Cell Number (x 10^4 cells/mL	Day 15 Astaxanthin (mg/L)
Control	3.93 ± 0.34	4.73 ± 0.25	3.87 ± 0.25	2.67 ± 0.17	1.47 ± 0.19	1.67 ± 0.25	0.190 ± 0.010
Paromomycin (0.25 g/L)	0.07 ± 0.09	0.27 ± 0.09	0.27 ± 0.09	0.33 ± 0.05	0.40 ± 0.16	0.27 ± 0.25	0.002 ± 0.002
Paromomycin (0.5 g/L)	0.07 ± 0.09	0.07 ± 0.09	0.40 ± 0.16	0.40 ± 0.08	0.40 ± 0.00	0.00 ± 0.00	0.006 ± 0.003
Paromomycin (1 g/L)	0.13 ± 0.09	0.00 ± 0.00	0.00 ± 0.00	0.00 ± 0.00	0.00 ± 0.00	0.13 ± 0.09	0.012 ± 0.002

Table S5. Cell numbers of *H. lacustris* grown in JM without nitrogen source (Day 1, Day 3, Day 6, Day 9, Day 12, Day 15) and astaxanthin content (Day 15) with the use of Tetracycline (0.25 g/L, 0.5 g/L, 1 g/L). Data are presented as mean ± standard deviation. Lowercase letters indicate significant differences ($p < 0.001$).

H. lacustris culture grown with Tetracycline	Day 1 Cell Number (x 10^4 cells/mL)	Day 3 Cell Number (x 10^4 cells/mL)	Day 6 Cell Number (x 10^4 cells/mL)	Day 9 Cell Number (x 10^4 cells/mL)	Day 12 Cell Number (x 10^4 cells/mL)	Day 15 Cell Number (x 10^4 cells/mL	Day 15 Astaxanthin (mg/L)
Control	3.93 ± 0.34	4.73 ± 0.25	3.87 ± 0.25^{b}	2.67 ± 0.17	1.47 ± 0.19	1.67 ± 0.736	0.190 ± 0.010^{b}
Tetracycline (0.25 g/L)	2.40 ± 0.16	1.40 ± 0.00	2.73 ± 0.25^{a}	2.13 ± 0.05	1.53 ± 0.19	0.40 ± 0.28	0.008 ± 0.003^{a}
Tetracycline (0.5 g/L)	1.60 ± 0.16	1.87 ± 0.25	2.47 ± 0.25^{ab}	1.90 ± 0.08	1.33 ± 0.09	1.33 ± 0.19	0.011 ± 0.003^{ab}
Tetracycline (1 g/L)	3.27 ± 0.09	1.33 ± 0.09	2.80 ± 0.33^{a}	2.07 ± 0.08	1.33 ± 0.09	2.07 ± 0.09	0.009 ± 0.002^{b}

Table S6. Cell numbers of *H. lacustris* grown in JM without nitrogen source (Day 1, Day 3, Day 6, Day 9, Day 12, Day 15) and astaxanthin content (Day 15) with the use of Kanamycin (0.25 g/L, 0.5 g/L, 1 g/L). Data are presented as mean ± standard deviation.

H. lacustris culture grown with Kanamycin	Day 1 Cell Number (x 10^4 cells/mL)	Day 3 Cell Number (x 10^4 cells/mL)	Day 6 Cell Number (x 10^4 cells/mL)	Day 9 Cell Number (x 10^4 cells/mL)	Day 12 Cell Number (x 10^4 cells/mL)	Day 15 Cell Number (x 10^4 cells/mL	Day 15 Astaxanthin (mg/L)
Control	3.93 ± 0.34	4.73 ± 0.25	3.87 ± 0.25	2.67 ± 0.17	1.47 ± 0.19	1.67 ± 0.25	0.190 ± 0.010
Kanamycin (0.25 g/L)	1.27 ± 0.09	0.07 ± 0.09	0.07 ± 0.09	0.03 ± 0.05	0.00 ± 0.00	0.00 ± 0.00	0.00 ± 0.000
Kanamycin (0.5 g/L)	0.40 ± 0.00	0.07 ± 0.09	0.13 ± 0.19	0.07 ± 0.09	0.00 ± 0.00	0.00 ± 0.00	0.00 ± 0.000
Kanamycin (1 g/L)	0.00 ± 0.00	0.00 ± 0.00	0.13 ± 0.09	0.07 ± 0.05	0.00 ± 0.00	0.00 ± 0.00	0.00 ± 0.000

Table S7. Cell numbers of *H. lacustris* grown in JM without nitrogen source (Day 1, Day 3, Day 6, Day 9, Day 12, Day 15) and astaxanthin content (Day 15) with the use of Chloramphenicol (0.25 g/L, 0.5 g/L, 1 g/L). Data are presented as mean ± standard deviation. Data are presented as mean ± standard deviation. Lowercase letters indicate significant differences ($p < 0.001$).

H. lacustris culture grown with Chloramphenicol	Day 1 Cell Number (x 10^4 cells/mL)	Day 3 Cell Number (x 10^4 cells/mL)	Day 6 Cell Number (x 10^4 cells/mL)	Day 9 Cell Number (x 10^4 cells/mL)	Day 12 Cell Number (x 10^4 cells/mL)	Day 15 Cell Number (x 10^4 cells/mL	Day 15 Astaxanthin (mg/L)
Control	3.93 ± 0.34	4.73 ± 0.25	3.87 ± 0.25	2.67 ± 0.17[b]	1.47 ± 0.19[b]	1.67 ± 0.25[b]	0.190 ± 0.010[b]
Chloramphenicol (0.25 g/L)	0.33 ± 0.09	0.53 ± 0.09	0.87 ± 0.09	2.20 ± 0.09[b]	3.53 ± 0.34[c]	2.33 ± 0.19[b]	0.278 ± 0.006[c]
Chloramphenicol (0.5 g/L)	0.00 ± 0.00	0.20 ± 0.00	0.07 ± 0.09	0.30 ± 0.09[a]	0.53 ± 0.19[ab]	0.07 ± 0.09[a]	0.00 ± 0.000[a]
Chloramphenicol (1 g/L)	0.00 ± 0.00	0.00 ± 0.00	0.00 ± 0.00	0.03 ± 0.05[a]	0.07 ± 0.09[a]	0.00 ± 0.00[a]	0.00 ± 0.000[a]

Table S8. Cell numbers of *H. lacustris* grown in JM without nitrogen source (Day 1, Day 3, Day 6, Day 9, Day 12, Day 15) and astaxanthin content (Day 15) with the use of Streptomycin (0.25 g/L, 0.5 g/L, 1 g/L). Data are presented as mean ± standard deviation.

H. lacustris culture grown with Streptomycin	Day 1 Cell Number (x 10^4 cells/mL)	Day 3 Cell Number (x 10^4 cells/mL)	Day 6 Cell Number (x 10^4 cells/mL)	Day 9 Cell Number (x 10^4 cells/mL)	Day 12 Cell Number (x 10^4 cells/mL)	Day 15 Cell Number (x 10^4 cells/mL	Day 15 Astaxanthin (mg/L)
Control	3.93 ± 0.34	4.73 ± 0.25	3.87 ± 0.25	2.67 ± 0.17	1.47 ± 0.19	1.67 ± 0.25	0.190 ± 0.010
Streptomycin (0.25 g/L)	0.13 ± 0.09	0.20 ± 0.16	0.13 ± 0.09	0.10 ± 0.08	0.07 ± 0.09	0.00 ± 0.00	0.011 ± 0.002
Streptomycin (0.5 g/L)	0.07 ± 0.09	0.00 ± 0.00	0.20 ± 0.16	0.13 ± 0.09	0.07 ± 0.09	0.00 ± 0.00	0.005 ± 0.000
Streptomycin (1 g/L)	0.20 ± 0.00	0.00 ± 0.00	0.00 ± 0.00	0.00 ± 0.00	0.00 ± 0.00	0.00 ± 0.00	0.00 ± 0.000

Table S9. Cell numbers of *H. lacustris* grown in JM without nitrogen source (Day 1, Day 3, Day 6, Day 9, Day 12, Day 15) and astaxanthin content (Day 15) with the use of Dihydrostreptomycin sulfate (0.25 g/L, 0.5 g/L, 1 g/L). Data are presented as mean ± standard deviation.

H. lacustris culture grown with Dihydrostreptomycin sulfate	Day 1 Cell Number (x 10^4 cells/mL)	Day 3 Cell Number (x 10^4 cells/mL)	Day 6 Cell Number (x 10^4 cells/mL)	Day 9 Cell Number (x 10^4 cells/mL)	Day 12 Cell Number (x 10^4 cells/mL)	Day 15 Cell Number (x 10^4 cells/mL	Day 15 Astaxanthin (mg/L)
Control	3.93 ± 0.34	4.73 ± 0.25	3.87 ± 0.25	2.67 ± 0.17	1.47 ± 0.19	1.67 ± 0.25	0.190 ± 0.010
Dihydrostreptomycin sulfate (0.25 g/L)	0.07 ± 0.09	0.07 ± 0.09	0.00 ± 0.00	0.03 ± 0.05	0.07 ± 0.09	0.00 ± 0.00	0.00 ± 0.000
Dihydrostreptomycin sulfate (0.5 g/L)	0.07 ± 0.09	0.00 ± 0.00	0.00 ± 0.00	0.00 ± 0.00	0.00 ± 0.00	0.00 ± 0.00	0.00 ± 0.000
Dihydrostreptomycin sulfate (1 g/L)	0.00 ± 0.00	0.00 ± 0.00	0.07 ± 0.09	0.03 ± 0.05	0.00 ± 0.000	0.00 ± 0.000	0.004 ± 0.003

Table S10. Cell numbers of *H. lacustris* grown in JM without nitrogen source (Day 1, Day 3, Day 6, Day 9, Day 12, Day 15) and astaxanthin content (Day 15) with the use of Penicillin (0.25 g/L, 0.5 g/L, 1 g/L). Data are presented as mean ± standard deviation. Lowercase letters indicate significant differences ($p < 0.001$).

H. lacustris culture grown with Penicillin	Day 1 Cell Number (x 10^4 cells/mL)	Day 3 Cell Number (x 10^4 cells/mL)	Day 6 Cell Number (x 10^4 cells/mL)	Day 9 Cell Number (x 10^4 cells/mL)	Day 12 Cell Number (x 10^4 cells/mL)	Day 15 Cell Number (x 10^4 cells/mL	Day 15 Astaxanthin (mg/L)
Control	3.93 ± 0.34	4.73 ± 0.25[a]	3.87 ± 0.25[a]	2.67 ± 0.17[a]	1.47 ± 0.19[a]	1.67 ± 0.25[a]	0.190 ± 0.010[a]
Penicillin (0.25 g/L)	3.33 ± 0.09	5.53 ± 0.25[a]	4.00 ± 0.33[a]	3.23 ± 0.25[a]	2.47 ± 0.09[a]	3.8 ± 0.09[c]	0.26 ± 0.003[b]
Penicillin (0.5 g/L)	2.53 ± 0.19	11.93 ± 0.25[b]	8.53 ± 0.25[b]	7.13 ± 0.21[b]	5.73 ± 0.19[b]	6.00 ± 0.28[b]	1.271 ± 0.007[d]
Penicillin (1 g/L)	3.27 ± 0.09	6.40 ± 0.16[a]	4.00 ± 0.33[a]	3.23 ± 0.33[a]	2.47 ± 0.09[a]	2.20 ± 0.16[a]	0.326 ± 0.004[c]

Table S11. Cell numbers of *H. lacustris* grown in JM without nitrogen source (Day 1, Day 3, Day 6, Day 9, Day 12, Day 15) and astaxanthin content (Day 15) with the use of Gentamycin (0.25 g/L, 0.5 g/L, 1 g/L). Data are presented as mean ± standard deviation.

H. lacustris culture grown with Gentamycin	Day 1 Cell Number (x 10^4 cells/mL)	Day 3 Cell Number (x 10^4 cells/mL)	Day 6 Cell Number (x 10^4 cells/mL)	Day 9 Cell Number (x 10^4 cells/mL)	Day 12 Cell Number (x 10^4 cells/mL)	Day 15 Cell Number (x 10^4 cells/mL	Day 15 Astaxanthin (mg/L)
Control	3.93 ± 0.34	4.73 ± 0.25	3.87 ± 0.25	2.67 ± 0.17	1.47 ± 0.19	1.67 ± 0.25	0.190 ± 0.010
Gentamycin (0.25 g/L)	0.27 ± 0.09	0.13 ± 0.09	0.00 ± 0.000	0.03 ± 0.05	0.07 ± 0.09	0.13 ± 0.19	0.00 ± 0.000
Gentamycin (0.5 g/L)	0.07 ± 0.09	0.00 ± 0.000	0.00 ± 0.000	0.03 ± 0.05	0.07 ± 0.09	0.07 ± 0.09	0.00 ± 0.000
Gentamycin (1 g/L)	0.07 ± 0.09	0.07 ± 0.09	0.00 ± 0.00	0.00 ± 0.00	0.00 ± 0.00	0.00 ± 0.00	0.00 ± 0.000
